# Superficial Cervical Plexus Block for Postoperative Pain Management in Occipital Craniotomies: A Narrative Review

**DOI:** 10.3390/medsci13030101

**Published:** 2025-07-28

**Authors:** Shahab Ahmadzadeh, Bennett M. Ford, Alex V. Hollander, Mary Kathleen Luetkemeier, Tomasina Q. Parker-Actlis, Sahar Shekoohi

**Affiliations:** 1 Department of Anesthesiology, Louisiana State University Health Sciences Center at Shreveport, Shreveport, LA 71103, USA; 2 School of Medicine, Louisiana State University Health Sciences Center New Orleans, New Orleans, LA 70112, USA; bford8@lsuhsc.edu; 3 School of Medicine, Louisiana State University Health Sciences Center at Shreveport, Shreveport, LA 71103, USAmhl001@lsuhs.edu (M.K.L.)

**Keywords:** occipital craniotomy, superficial cervical plexus block, regional anesthesia, postoperative pain, opioid-sparing, scalp block, neurosurgery

## Abstract

Post-craniotomy pain is common yet often sub-optimally managed because systemic opioids can obscure postoperative neurologic examinations. The superficial cervical plexus block (SCPB) has, therefore, emerged as a targeted regional anesthesia option for occipital craniotomies. The SCPB targets the C2–C4 nerves to anesthetize the occipital scalp region, covering the lesser occipital nerve territory that lies within typical posterior scalp incisions. Clinical evidence shows the block is effective in reducing acute postoperative pain after occipital craniotomy and diminishes opioid requirements. Studies have demonstrated successful and long-lasting analgesia, reductions in 24-h opioid consumption, and a lower incidence of severe pain. Moreover, the technique exhibits a low complication rate and is safer than a deep cervical plexus block because the injection remains superficial and avoids critical vascular and neural structures. When delivered under ultrasound guidance, major adverse events are exceedingly rare. By reducing opioid use, the SCPB can help reduce postoperative complications, allowing earlier neurological assessments and fewer opioid-related side effects. Incorporation of the SCPB into multimodal analgesia regimens can, therefore, accelerate postoperative recovery by providing regionally focused, opioid-sparing pain control without clinically significant sedation. Overall, current data support the SCPB as a dependable, well-tolerated, and clinically practical approach for managing post-craniotomy pain in patients undergoing occipital approaches. In this narrative review, we will discuss the mechanism of action and anatomy, the clinical application, safety and tolerability, patient outcomes, and emerging future directions of the superficial cervical plexus block and how it mitigates post-occipital craniotomy pain.

## 1. Introduction

Post-craniotomy pain remains a prevalent yet inconsistently treated problem, with up to two-thirds of patients reporting moderate to severe discomfort in the first postoperative days [1]. With opioids being the frontline treatment post-operation, clinicians often undertreat them because systemic opioids can depress consciousness, confound neurologic examination, and provoke respiratory depression, nausea, or vomiting [1]. Uncontrolled nociceptive input can trigger sympathetic surges, raising intracranial and arterial pressures and risking hematoma formation, while inadequate relief may delay mobilization [1]. Moreover, persistent incisional headache develops in up to 50% of survivors, impairing quality of life and increasing long-term healthcare utilization [2].

The posterior scalp incised during occipital or suboccipital craniotomy receives cutaneous innervation predominantly from the ventral rami of C1–C4, carried by the lesser occipital, great auricular, transverse, and supraclavicular cervical nerves [3,4]. An ultrasound-guided superficial cervical plexus block (the SCPB) deposits local anesthetic at the posterior border of the sternocleidomastoid, bathing these branches without entering the deeper cervical paravertebral space [3]. With the patient supine and the head turned contralateral to the side of the block, the SCPB can be performed within five minutes, making it a simple and easy addition to routine neurosurgical preparation [4,5]. By targeting only the cutaneous branches, the SCPB renders precise anatomical areas of the head, face, and anterior aspect of the neck insensible and painless, while avoiding phrenic nerve injury and other deep block complications [3,4].

Clinical evidence substantiates its effectiveness. In the first randomized trial, Girard et al. (2010) demonstrated that bilateral the SCPB administered at skin closure produced pain scores and rescue-analgesic timing equivalent to intravenous morphine yet avoided high systemic opioid doses [6]. More contemporary data are even more compelling: Zeng et al. (2022) found that a pre-incision ultrasound-guided the SCPB nearly halved 24-h patient-controlled opioid requirements and reduced severe pain from 26% to 7% [5]. The opioid consumption of sufentanil was significantly lower in the first 24 h, and the total perioperative consumption was significantly reduced as well, allowing hemodynamic stability [5]. The longer-term benefit is also possible—an expanded cohort showed a 34% versus 51% prevalence of persistent incisional pain three months after suboccipital craniotomy when the SCPB was used [2].

An SCPB is generally regarded as a safe procedure, with multiple trials and case series reporting a low incidence of block-related complications. Because injection remains superficial, the SCPB avoids the vertebral artery and phrenic nerve, differentiating it from deep cervical plexus techniques that can cause intravascular or diaphragmatic complications. Across contemporary series, major adverse events are exceedingly rare, and ultrasound guidance further mitigates risk [5]. This safety profile makes the SCPB a solid adjunct in multimodal analgesia protocols, such as when pairing regional blocks with acetaminophen, non-steroidal anti-inflammatory drugs, and low-dose opioids [5].

In summary, post-occipital craniotomy pain is a significant problem, yet it can be managed better when the SCPB is implemented. The ultrasound-guided the SCPB offers a targeted method to block the sensory innervation of the posterior scalp, providing therapeutic analgesia while avoiding many drawbacks of systemic opioids. The SCPB thus helps avoid many common postoperative pain complications that follow post-occipital craniotomies.

## 2. Mechanism of Action and Anatomy

The superficial cervical plexus block (the SCPB) achieves postoperative analgesia by anesthetizing the superficial sensory branches of the cervical plexus—chiefly C2 to C4—thereby interrupting nociceptive input from the posterior scalp and upper neck [7]. Anatomically, the cervical plexus originates from the anterior rami of C2 to C4, with its four cutaneous terminal branches—the lesser occipital, great auricular, transverse cervical, and supraclavicular nerves, emerging at the midpoint of the posterior border of the sternocleidomastoid (SCM) muscle, also known as “Erb’s point”[7]. The SCPB specifically targets the lesser occipital (C2–C3) and great auricular (C2–C3) nerves, which innervate the upper and posterior aspect of the scalp, auricle, and mastoid region—sites directly involved in occipital craniotomy incisions [2,6]. The block is administered subcutaneously along the posterior SCM border, which bathes these superficial branches after they pierce the investing fascia [5,8].

Ultrasound guidance further refines this technique by visualizing the depot in the subcutaneous plane superficial to the SCM, avoiding deeper penetration and minimizing associated risks [7,8]. Injection at this anatomic locus allows anesthetic diffusion to reach the lesser occipital and great auricular nerves before they traverse into the scalp and auricular regions, blocking the transmission of nociceptive signals from surgical incisions involving the occipital bone and upper neck [7]. By preventing these afferent signals from reaching the dorsal horn, the SCPB mitigates central sensitization, reduces postoperative pain intensity, and decreases opioid requirements—a mechanism supported by clinical studies demonstrating its efficacy in occipital and infratentorial craniotomies [2,6].

The lesser occipital nerve originates from the ventral rami of C2 (and occasionally C3), passes anterior to the spinal accessory nerve, and ascends along the posterior SCM to perforate the deep fascia, supplying sensory innervation to the postauricular scalp. The great auricular nerve, arising from the ventral rami of C2–C3, ascends vertically across the SCM, innervating the skin over the parotid region, auricle, and mastoid area [7]. The SCPB’s anatomical precision ensures the blockade of both nerves at their emergence points, where their sensory distribution directly relates to occipital craniotomy incisions [2,5].

This block’s mechanism of action hinges on its interruption of peripheral nociceptive afferent signaling within the C2–C4 dermatomes before those impulses reach the spinal cord. Local anesthetics work by blocking action potentials in tissue via inhibition of Na+ channels, thus resulting in inhibition of nociceptive fibers and pain transmission signals [9]. These nociceptive fibers are A-delta fibers, lightly myelinated and responsible for the initial perception of pain, and C-fibers, more myelinated and relay pain intensity [10]. By delivering local anesthetic—typically bupivacaine, ropivacaine, levobupivacaine, or lidocaine—into the subplatysmal fascial plane near the posterior border of the SCM, the SCPB halts signal propagation in A-delta and C-fiber pathways [2,3,9]. This blockade not only reduces local inflammatory hyperalgesia but may also diminish central sensitization if performed preemptively [11]. Furthermore, the anatomic predictability of the target nerves’ emergence from the posterior SCM border makes the SCPB reproducible and safe. The superficial nature of the injection minimizes risk while ensuring sufficient analgesia across the occipital and lateral neck dermatomes. 

## 3. Clinical Application and Efficacy

Superficial cervical plexus block has emerged as a clinically successful regional anesthesia technique for occipital craniotomies, providing analgesia comparable to systemic opioids while significantly reducing postoperative opioid consumption and delaying the need for rescue analgesia. In a randomized, double-blind trial of patients undergoing infratentorial or occipital craniotomy, bilateral the SCPB using 0.5% bupivacaine/2% lidocaine provided transitional analgesia clinically equivalent to intravenous morphine (0.1 mg/kg) immediately postoperatively, with no significant difference in numerical rating scale (NRS) pain scores (~3.9 vs. 4.3) or time to first codeine rescue dose (median 25 vs. 21.5 min) [6]. This equivalence in analgesic quality suggests the SCPB is a reliable opioid-sparing alternative with comparable efficacy and fewer systemic side effects. 

Building on this evidence, a larger, prospective randomized trial in suboccipital retrosigmoid craniotomy patients demonstrated marked opioid-sparing benefits. In this study, preoperative ultrasound-guided the SCPB using 0.5% ropivacaine reduced 24-h sufentanil consumption by nearly 50% (5.0 µg vs. 9.8 µg via patient-controlled intravenous analgesia; *p* = 0.001) and decreased total perioperative sufentanil use (45.0 µg vs. 54.5 µg; *p* = 0.001) [5]. Furthermore, the incidence of severe postoperative pain dropped significantly (7.5% vs. 26.4%), accompanied by a reduction in the area under the pain score curve from 1 to 48 h, indicating sustained analgesic efficacy [5].

In terms of delayed rescue analgesia, studies have shown that the SCPB extended the time to the first opioid dose. The occipital craniotomy trial reported comparable rescue timing to morphine, suggesting noninferiority, while the larger sufentanil study demonstrated not only reduced consumption but also delayed analgesic demand, suggesting diminished central sensitization and prolonged nociceptive blockade [5,6].

Mechanistically, the SCPB interrupts sensory transmission in A-delta and C-fibers within the C2–C4 dermatomes, specifically targeting the lesser occipital and great auricular nerves at their emergence from the posterior border of the SCM. This interruption reduces nociceptive input before it can engage central sensitization pathways, contributing to both acute and sustained postoperative pain control [12]. 

Clinically, the opioid-sparing effects of the SCPB are significant: the nearly halved opioid consumption translates to fewer opioid-related adverse effects—nausea, vomiting, respiratory depression—potentially improving recovery and reducing hospitalization burden. Moreover, early equivalency to morphine without the need for systemic opioids suggests the SCPB is especially valuable when opioid side effects could hinder neurological monitoring or recovery [5,6]. 

While scalp blocks remain a more common regional analgesic technique in craniotomies, guidelines from recent systematic reviews continue to advocate for regional methods in general, although the SCPB is not yet specifically highlighted. Nonetheless, evidence is accumulating that the SCPB offers analgesic benefits similar to scalp blockade and is particularly promising for occipital approaches [13].

To summarize, the SCPB delivers opioid-comparable analgesia, significantly reduces postoperative opioid requirements, and delays rescue analgesic needs in occipital and infratentorial craniotomies. With its targeted interruption of C2–C4 sensory fibers and demonstrated clinical efficacy, the SCPB presents a compelling opioid-sparing strategy with a favorable safety profile, particularly suitable in neurosurgical settings where minimizing systemic opioid exposure is critical. 

## 4. Safety and Tolerability

Superficial cervical plexus block stands out as a technically straightforward procedure, employing a single superficial subcutaneous injection along the posterior border of the sternocleidomastoid to target the sensory branches of C2–C4 [7]. This technique contrasts sharply with deep cervical plexus blocks, which necessitate needle advancement to the transverse processes and deeper fascial layers—steps that inherently increase procedural complexity and risk [14]. The SCPB can be performed reliably using either a landmark-guided or ultrasound-assisted approach, requiring minimal training and consistently demonstrating low complication rates [12].

Because the SCPB confines anesthetic deposition to the subcutaneous tissue superficial to the investing fascia of SCM, it avoids intrusion into the carotid sheath, vertebral artery, or neuraxial spaces [12]. MRI-confirmed fascial compartmentalization supports this limited spread, which protects against epidural or subarachnoid diffusion events more commonly reported in deep cervical plexus blocks [12,15]. Moreover, randomized trials indicate that hemi-diaphragmatic paralysis—a hallmark of phrenic nerve blockade—occurs rarely following superficial blocks, whereas it affects a substantial proportion of patients receiving deep CPBs (diaphragmatic excursion 2.04 cm vs. 4.34 cm for superficial blocks; *p* < 0.001) [12].

Notably, neurosurgical trials involving an SCPB in occipital and infratentorial craniotomies have reported no major complications, aligning with larger meta-analyses of scalp nerve blocks that found a low incidence of adverse events across over a dozen randomized trials. These systematic reviews confirmed a lack of increased risk for nerve injury, hematoma, or local anesthetic systemic toxicity (as well as equivalent complication rates in pre- vs. post-incision scalp blocks), underscoring the safety of regional blocks in craniotomy patients [13].

The superficial technique also eliminates major concerns associated with phrenic nerve involvement, vertebral artery injury, or neuraxial spread—risks demonstrably higher with deeper blocks—with diaphragmatic motion and pulmonary function preserved following the SCPB [12]. While rare occurrences of transient issues like Horner’s syndrome or minor hematoma have been reported, these are self-limited and notably infrequent following SCPB compared with deep or combined blocks [16].

In contrast, scalp blocks—although generally low risk—require multiple injection sites and involve more vascular tissues, which can introduce rare but serious complications like intracranial bleeding or increased systemic anesthetic absorption [13,17]. the SCPB avoids these concerns by requiring only one low-risk injection site, with minimal vascularity and reduced anesthetic volumes. Evidence from emergency and perioperative settings confirms both its tolerability and broad suitability, even in patients with high comorbidity burden [14,15].

In terms of anesthetic drug selection, concentration, and dosage, bupivacaine is the most frequently reported agent for the SCPB [4]. The concentration varies (0.25% and 0.5%) as well as the volume injected, which is between 5 to 10 mL; however, only a few cases in the literature were less than 5 mL or between 10 and 15 mL [4]. Other drugs such as ropivacaine and levobupivacaine are used, but less commonly, and dosing and concentrations remain similar (0.5% with concentrations ranging from 10 mL to 20 mL) [2,4,18]. The duration in past studies has found ropivacaine to provide less effective analgesia, only lasting 4–8 h after a single injection, which is not adequate for longer surgeries and requires adjuncts such as dexmedetomidine [18]. However, when ropivacaine was used in conjunction with dexmedetomidine, the analgesia was extended and comparable to other local anesthetics [18].

Pharmacologic considerations favor ropivacaine over racemic bupivacaine in patients at higher risk for local anesthetic systemic toxicity due to consistently demonstrated lower cardiovascular and CNS toxicity thresholds in experimental and comparative studies [19]. Research has shown that CNS toxicity is reached when plasma levels reach 0.1 to 0.2 mg/L for unbound ropivacaine, bupivacaine, and levobupivacaine [20]. Yet studies show that in adult volunteers receiving continuous infusions, ropivacaine has higher tolerated doses and greater unbound concentrations before CNS toxicity than bupivacaine, suggesting again a better safety profile in toxicity [20]. Overall, current evidence indicates agent and volume selection for superficial blocks is individualized. Bupivacaine remains predominant for prolonged analgesia without adjuncts needed, while ropivacaine offers a lower toxicity alternative whose duration can be extended with dexmedetomidine or dexamethasone.

There are contraindications for an SCPB, and caution is warranted for its use in these cases. Most of the local anesthetic, such as ropivacaine or bupivacaine, that are given during the SCPB is cleared from the liver [21]. As such, individuals who have intrinsic hepatic disease and reduced hepatic blood flow are at a higher risk of a metabolite buildup and systemic toxicity [21]. However, patients with liver disease can still receive a single-shot block with a normal dose; only if a repeat block of ropivacaine and bupivacaine is performed does it need to be significantly reduced (10–50%) [22]. As the kidney is also key in the clearance of metabolites, renal impairment is an important consideration when using local anesthetics [23]. Usually, kidney dysfunction does not increase the risk of a potential systemic toxicity from local anesthetics, unless there is already metabolic dysfunction present, such as hypoxia or acidosis [23]. With that being said, patients with advanced kidney failure can have increased absorption of these anesthetics and thus reach peak concentrations quicker, necessitating more monitoring and an assessment of a lower dosage to achieve the same analgesia without causing systemic toxicity [23]. Patients with heart failure are also at an increased risk of systemic toxicity; however, the mechanism remains unclear if this is due to hepatic congestion, renal impairment, or reduced absorption capability, and physicians should follow the safe dosage of the anesthetic being used [23]. Patients in mild cardiac failure may not need to change the dose for single-shot blocks, but, similar to patients with liver disease, they require a dose reduction for repeat blocks to avoid systemic toxicity [21]. A systemic local anesthetic toxicity often presents with neurological signs such as blurry vision, perioral tingling, slurred speech, and drowsiness, as well as cardiovascular signs such as hypertension and tachycardia [24]. Close monitoring is required to evaluate local anesthetic systemic toxicity (LAST), with first-line treatment now being lipid emulsion as well as high-dose epinephrine [24].

The most common concern with the SCPB is for patients who have paralysis of the contralateral phrenic nerve and diaphragm or those who have had previous neck or radiation surgeries [4,25]. Complications arising from the SCPB are sparse, and again, while the SCPB avoids deeper structures, there can be complications that more commonly arise when the injectate spreads into the deep cervical plexuses by accident [4,25]. The most commonly reported complication when this occurs is paralysis of the ipsilateral phrenic nerve and, less commonly, recurrent laryngeal nerve paresis [4]. Other complications are local infection in the injection site, an allergy to the anesthetic that is used, hematoma, transient ischemic attack, or local anesthetic systemic toxicity as previously mentioned [4,7].

The SCPB is generally a well-tolerated block with a shallow technical profile and minimal risk. Its superficial execution avoids deep neck structures and phrenic nerve compromise, reducing the potential for serious complications. Trials in craniotomy contexts and scalp block meta-analyses corroborate its clean safety profile, making the SCPB a preferable alternative to deeper cervical plexus or scalp blocks, especially in neurosurgical patients where safety and simplicity are paramount. See Table 1 below.

## 5. Patient Outcomes and Practice Considerations

Superficial cervical plexus blocks (SCPBs) are emerging as a practical and valuable tool in postoperative occipital craniotomy patients. Several emerging studies highlight the benefits of peripheral nerve blocks in conjunction with other analgesic therapies. By providing targeted analgesia to the posterior scalp and neck, the SCPBs can help reduce the need for systemic opioids and the range of side effects that often come with them, such as nausea, constipation, sedation, and respiratory depression [26,27]. These side effects can become life-threatening in higher-risk patients, such as the elderly, patients with sleep apnea, patients who are obese, and patients with a history of smoking [27]. Postoperative regional anesthesia and other analgesic techniques can result in lower patient pain scores compared to when systemic opioids are used and reduce the incidence of postoperative chronic pain [28]. It was also seen that these patients had reduced opioid side effects through the opioid-sparing effect [28].

Several recent studies support the role of the SCPBs in improving pain control while minimizing opioid use. A randomized trial by Girard et al. (2010) found the SCPBs to be just as effective as morphine in patients undergoing infratentorial craniotomies, with no increase in side effects [6]. A randomized controlled trial by Zeng et al. (2022) showed that the SCPB does provide substantial pain management in patients who underwent craniotomies and tumor resections via the suboccipital retrosigmoid approach [5]. This study also found that the SCPB has an opioid-sparing effect, and patients who received the SCPB before suboccipital craniotomy required less opioid medication and experienced less severe pain overall [5]. In a 2025 follow-up study, Zeng and colleagues reported a lower incidence of incisional pain at three months in patients who received a block compared to those who did not [2]. 

In addition to these clinical benefits, the SCPBs are associated with greater patient satisfaction. This likely reflects both the improved pain relief at rest and with movement and the avoidance of side effects that patients commonly find distressing [27]. Evidence from thyroid and head and neck surgeries suggests that patients receiving the SCPBs report better overall experiences, with fewer interruptions to comfort or function [29,30]. Studies have also found that the SCPBs are relatively beneficial and simple to implement. When performed under ultrasound guidance, the block can be placed quickly with minimal risk at the end of surgery without repositioning the patient or disrupting the operative timeline. This type of procedure falls under the category of POCUS (Point of Care Ultrasound) [31]. Currently, according to a recent survey in 2023 from chiefs of staff for Veterans Affairs hospitals, the main barrier to using POCUS is inadequate funding (35%), a shortage of trained providers (28%), and a lack of training in general (33%) [32]. More training and resources should be given for using POCUS procedures such as the SCPB, as the advantages are widespread. Some main advantages include lower dosage and faster onset of action [33]. See Table 2 below. Furthermore, neck surgeries in a semi-urban area in India found the overall cost of the SCPB to be 10 times less expensive than general anesthesia, even when used in conjunction with other adjuncts such a ketamine [34]. As a cost comparison, the study compared the average minimal cost of drugs for general anesthesia (GA) and the average maximum cost of the SCPB and SCBP + ketamine. The study found the cost of drugs for GA to be approximately 2.8 times higher than the SCPB + ketamine and 3.7 times higher than the SCPB alone. GA also required costs of oxygen, volatile anesthetic agents, and a post operative recovery cost, which the the SCPB and the SCPB + ketamine groups did not. This post operative recovery stays included costs such as oxygen, oxygen delivery consumables, and a minimum one day stay. While this study looked at neck surgeries such as thyroidectomies and a comparison with general anesthesia, the cost-effectiveness of the SCPB is demonstrated [34]. Future studies should investigate the impact the SCPB has on cost effectiveness, such as reducing postoperative complications that arise from mismanaged pain, resulting in increased cost of care [35].

## 6. Future Directions and Emerging Research

Although early data on superficial cervical plexus blocks (the SCPBs) in neurosurgical patients are encouraging, there are still significant gaps in the research. Most of the published studies have been relatively small and conducted at single institutions [5,6]. To truly understand how successful the SCPBs are and in which settings they’ll need to be evaluated in larger, multicenter randomized controlled trials. There is currently an ongoing multicenter, randomized, placebo-controlled Phase 4 trial in Australia examining how the SCPB affects postoperative recovery in patients undergoing anterior cervical spine surgery. While this is investigating anterior cervical spine surgery, the findings will shed more light into the how the SCPB mitigates post operative pain from head and neck surgeries such as craniotomies. The last data collection date was 10 February 2022 [36]. Results are still pending. Another area that needs more research is the use of other methods to enhance block duration, possibly with the use of continuous infusion or catheter infusion [27,28]. Finally, while the SCPBs appear to reduce acute pain and early opioid use, their potential to influence long-term outcomes is still unclear. Although a study found that patients who received an the SCPB had lower rates of incisional pain three months after surgery, further research is needed to determine whether these benefits persist for a longer period [2]. 

## 7. Conclusions

As neurosurgical practices continue to evolve toward more patient-centered, recovery-focused care, the role of regional anesthesia, in this case, superficial cervical plexus blocks (the SCPBs), is being further investigated. For patients undergoing occipital craniotomy, postoperative pain can be challenging to manage due to the surgical site’s unique innervation and the need for clear neurologic assessment in the immediate recovery period. the SCPBs offer a targeted and safe block that can provide analgesia while minimizing the need for systemic opioids.

The first key advantage of the SCPBs is their ability to reduce opioid use and its associated side effects. Opioids remain the basis of postoperative pain management, but they are far from ideal, especially in neurosurgical patients, where sedation, respiratory depression, and nausea can interfere with both recovery and neurologic evaluation. the SCPBs help mitigate this problem by providing effective, localized pain relief that spares patients from the systemic burden of opioids. Secondly, the SCPBs have been shown to support faster recovery and improved patient experience. Studies have linked their use with earlier mobilization, greater patient comfort, and higher satisfaction due to the combination of pain relief and reduced side effects. These studies have also shown that the SCPBs can result in a shorter hospital stay for these postoperative patients. As patient-reported outcomes become more important in evaluating the quality of surgical care, these benefits make the SCPBs particularly relevant. Finally, the SCPBs are notable for their simplicity and ease of integration into existing neurosurgical workflows. When performed under ultrasound guidance, the block is technically straightforward, well-tolerated, and fast. It can be administered at the conclusion of surgery with minimal disruption to operative protocols. These practical advantages make it well-suited for routine use in both academic and community hospital settings. 

The current evidence supports the use of the SCPBs as a promising adjunct in the multimodal management of pain following occipital craniotomy. the SCPBs offer targeted, opioid-sparing analgesia and improve patient outcomes and satisfaction. They also implement easily into postoperative workflows. More studies are needed to validate and expand upon these findings, particularly in broader neurosurgical populations and with long-term follow-up; however, the SCPBs provide reliable postoperative analgesia and can be confidently incorporated into standard post-craniotomy care.

## Figures and Tables

**Table 1:** Comparative summary of superficial cervical plexus block, deep cervical plexus block, and scalp nerve block for craniotomy analgesia.

Feature	The SCPB	DCPB	Scalp Nerve Block
Target Nerves	C2–C4 superficial (lesser occipital, great auricular) [5,6]	Deep branches of C2–C4 [12]	Supraorbital, supratrochlear, auriculotemporal, occipital [13]
Injection Depth	Subcutaneous, posterior to SCM [6]	Deep to SCM, near transverse processes [12]	Subcutaneous at multiple sites [13]
Imaging Use	Optional (landmark or ultrasound) [7]	Ultrasound highly recommended [12]	Typically landmark-guided [13]
Analgesic Coverage	Occipital and postauricular regions [5,6]	Broad unilateral cervical region [12]	Entire scalp depending on completeness [13]
Technical Complexity	Simple and reproducible [7]	Moderate to high [12]	Moderate (requires 6+ injections) [13]
Risk of Phrenic Nerve Block	Very low [12]	Moderate to high [12]	None [13]
Risk of Neuraxial Spread	Negligible [12]	Possible [12]	None [13]
Adverse Events Reported	Rare; Horner’s, minor hematoma [16]	Phrenic palsy, epidural or vascular spread [12]	Rare; hematoma, LA toxicity [13,17]
Use in Occipital Craniotomy	Highly suitable [5]	Less preferred due to risks [12]	Effective but more complex [13]
Opioid-Sparing Effect	Demonstrated in RCTs [5]	Limited data [12]	Demonstrated in meta-analyses [13]

DCPB = deep cervical plexus block; LA = local anesthetic; RCT = randomized controlled trial; SCM = sternocleidomastoid muscle; SCPB = superficial cervical plexus block.

**Table 2:** Comparative Studies.

Author(s)/Study	Intervention	Results	Conclusion
Kehlet et al., 1993 [26]	“Multimodal” or “Balanced Analgesia,” including regional anesthesia	Reduced opioid use/side effects, improved recovery, possibly increased early mobilization, and reduced hospital stay	Regional techniques enhance recovery by reducing opioid needs
Rawal et al., 2016 [27]	Regional anesthesia for postoperative opioid reduction	Lower incidence of opioid-related side effects	Regional blocks are especially useful in high-risk populations
Wu et al., 2011 [28]	Peripheral nerve blocks as part of multimodal analgesia	Improved analgesia, reduced sedation, and opioid use	Multimodal strategies offer superior pain control and reduce complications, and improved outcomes in high-risk patients/procedures
Mayhew et al., 2018 [29]	Bthe SCPB in thyroid surgery (meta-analysis)	Reduced pain scores, shorter time to analgesia, shorter hospital stay	Bthe SCPB improves postoperative analgesia and expedites recovery
Ilfeld et al., 2011 [30]	Continuous peripheral nerve blocks	Better postoperative pain control and functional recovery	Peripheral nerve blocks enhance recovery across surgical specialties
Peng et al., 2020 [33]	Ultrasound-guided the SCPB in suboccipital retrosigmoid craniotomy (RCT protocol)	Outlined procedural design, safety protocol, and analgesia endpoints for the SCPB	Established safe, replicable method for the SCPB administration in neurosurgery trials
Girard et al., 2010 [6]	the SCPB vs. morphine for infratentorial craniotomy	Equivalent pain relief and rescue analgesic use; similar nausea/vomiting	the SCPB offers analgesia comparable to systemic opioids in neurosurgery
Zeng et al., 2022 [5]	Preoperative the SCPB (ropivacaine) vs. saline	Lower 24-h opioid use and reduced severe pain	the SCPB significantly reduces acute postoperative opioid needs
Zeng et al., 2025 [2]	the SCPB with ropivacaine vs. placebo for suboccipital craniotomy	Reduced persistent incisional pain at 3 months	the SCPB contributes to reduced long-term post-craniotomy pain

Bthe SCPB = Superficial Cervical Plexus Block; Bilateral the SCPB = Superficial Cervical Plexus Block; RCT = Randomized Controlled Trial.

## Data Availability

No new data were created or analyzed in this study.
